# Rolul Dietei Nesănătoase în Creșterea Incidenței de Hipertensiune Arterială în Rândul Populației 

**Published:** 2014

**Authors:** C Cazac

**Affiliations:** * Universitatea de Medicină și Farmacie “Carol Davila”, București

**Rezumat:**Sănătatea fizică și psihică bună reprezintă indubitabil unele dintre cele mai prețioase calități pe care le poate poseda o persoană. Fără aceste elemente, orice activitate simplă devine un efort iar orice efort devine o imposibilitate. Astfel, o mare parte din populația din România care se prezintă în camera cu gardă cu stare generală alterată dată de hipertensiunea arteriala (HA) raportează din ce în ce mai des un consum excesiv de alimente bogate în sare, zahăr sau grăsimi. Acest articol face o corelare directă între numărul crescut de cazuri de HA în România și creșterea numarului de persoane care au un regim alimentar nesănătos; analizează obiceiurile alimentare curente din România; și în final introduce metode prin care aceastea pot fi îmbunătățite pentru a reduce numărul de cazuri de HA. 

Cuvinte cheie: Regim Alimentar, Hipertensiune Arterială, IRES, Ateroscleroză, Diabet Zaharat

**Introducere**

**Ce reprezintă o dietă nesănătoasă?**

O dieta nesanatoasă fie [**1**] nu reușește să furnizeze organismului cantitățile de nutrienți în combinațiile necesare pentru o bună funcționare a acestuia sau [**2**] furnizează organismului o cantitate exagerată de clase alimentare de care acesta nu are nevoie [**5**]. Ambele cazuri se soldează cu apariția uneia sau a mai multor probleme medicale.

În continuare, un sondaj efectuat în perioada 14-15 mai, 2013, pe un eșantion de 1160 de persoane care aveau vârsta peste 18 ani a arătat că 91% dintre persoanele participante afirmă ca Românii manâncă nesănătos [**10**]. Acest număr mare evidențiază o predispoziție extraordinară spre dezvoltarea bolilor cardiovasculare printre care cea mai fregventă este HA.

**Incidența HA în România**

Într-adevăr, în cadrul congresului de nefrologie de la Sibiu organizat în perioada 24-26 Octombrie 2013, Președintele Societăţii Române de Hipertensiune Dr. Maria Dorobanțu a anunțat că *“Aproximativ 40% dintre Români suferă de HA iar mulți dintre ei nici nu știu despre aceasta deoarece se obișnuiesc cu simptomatologia ușoară”* [**11**-**13**].

Însă, lăsată netratată, pericolul cel mai mare al HA este că aceasta se poate dezvolta în afecțiuni mult mai grave. Unul dintre cele mai mari pericole ale HA este dezvoltarea cardiopatiei ischemice cu instalarea infarctului miocardic [**37**].

În anul 2010, România a fost pe primul loc dintre toate statele Uniunii Europene (UE) în ceea ce privește numărul de decese din cauza infarctului miocardic la bărbați cu un număr de 618 cazuri de decese din 100,000 bărbați [**15**]. De asemenea, România s-a situat pe locul 2 dintre toate statele membre ale UE la numărul de decese de cauza infarctului miocardic la femei înregistrând un număr de 412 decese la 100,000 femei [**15**]. În cazul numărului de decese la femei, România a fost precedată doar de Malta care a înregistrat 455 cazuri (o diferență de doar 43 cazuri între cele 2 state) [**15**].

**Alimentele consumate predominant și riscul dezvoltării HA:**

**Fig. 1:**
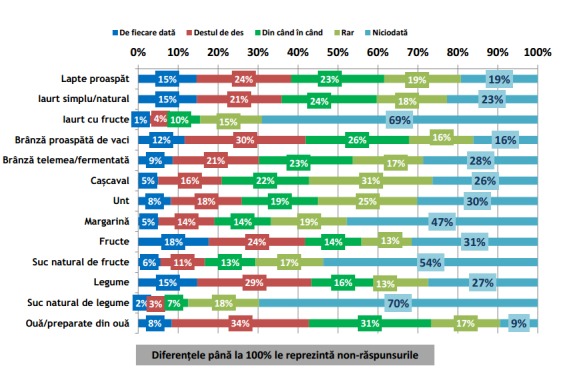
Incidența de consum a grupelor alimentare meționate în grafic în timpul micului dejun. Reprodus de pe pagina de web a IRES cu link-ul: http://www.ires.com.ro/uploads/articole/ires_obiceiurile_alimentare_ale_romanilor_2013.pdf

**Fig. 2:**
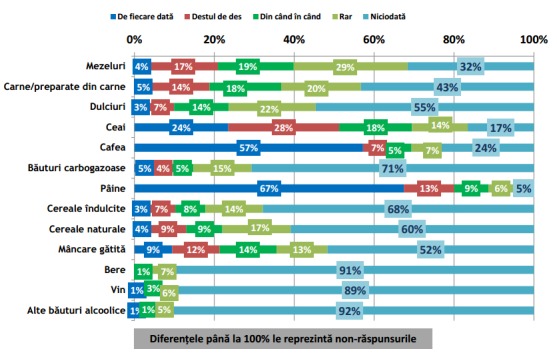
Incidența de consum a grupelor alimentare meționate în grafic în timpul micului dejun (Continuare). Reprodus de pe pagina IRES cu link-ul: http://www.ires.com.ro/uploads/articole/ires_obiceiurile_alimentare_ale_romanilor_2013.pdf

Conform rezultatelor sondajului IRES reprodus în **Fig. 1** și **Fig. 2**, se poate observa că în timpul micului dejun cele 5 alimente și băuturi preferențiale Românești sunt: (1) pâinea, (2) ouă și preparate din ouă, (3) cafeaua, (4) ceaiul și (5) brânza proaspătă de vaci [**10**]. Mai mult decât atât, păinea este alimentul cel mai mult consumat pe toată durata zilei **Fig. 1** – **Fig. 7**.

**Dieta Medie a Unui Român:**

**Fig. 3:**
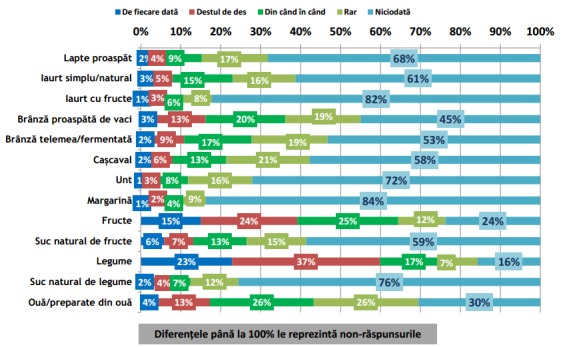
Incidența de consum a grupelor alimentare meționate în grafic în timpul Pranzului (Continuare). Reprodus de pe pagina IRES cu link-ul: http://www.ires.com.ro/uploads/articole/ires_obiceiurile_alimentare_ale_romanilor_2013.pdf

**Fig. 4:**
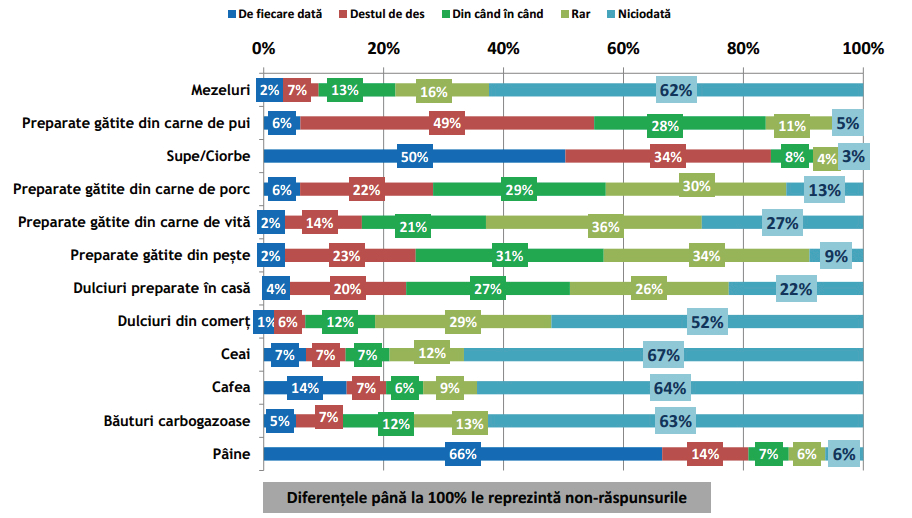
Incidența de consum a grupelor alimentare meționate în grafic în timpul Prânzului. Reprodus de pe pagina IRES cu link-ul: http://www.ires.com.ro/uploads/articole/ires_obiceiurile_alimentare_ale_romanilor_2013.pdf

**Fig. 5:**
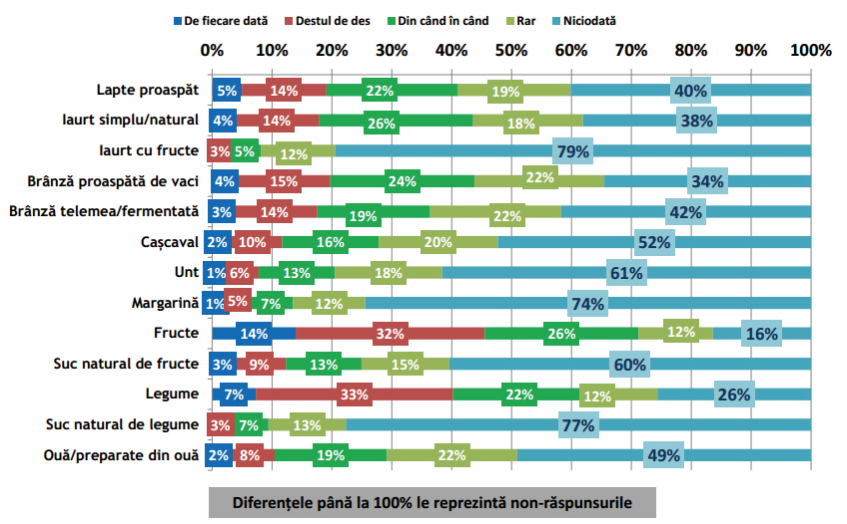
Incidența de consum a grupelor alimentare meționate în grafic în timpul Cinei. Reprodus de pe pagina IRES cu link-ul: http://www.ires.com.ro/uploads/articole/ires_obiceiurile_alimentare_ale_romanilor_2013.pdf

**Fig. 6:**
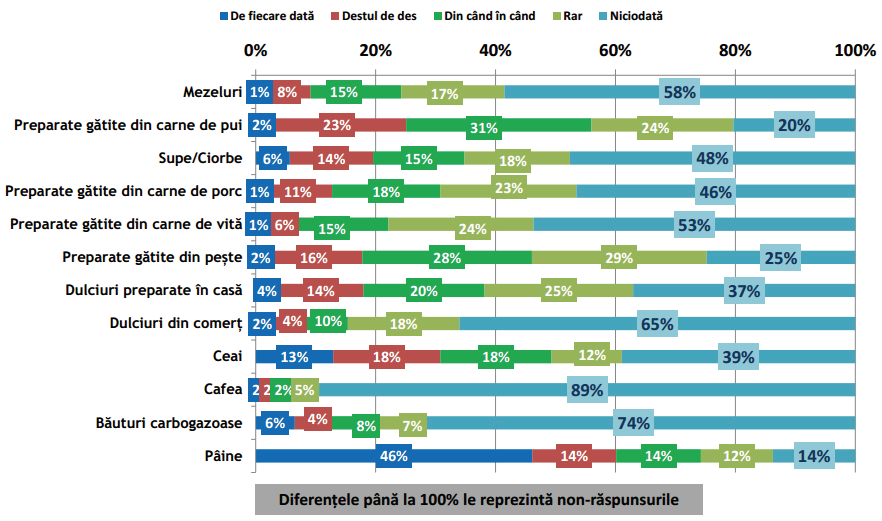
Incidența de consum a grupelor alimentare meționate în grafic în timpul Cinei (Continuare). Reprodus de pe pagina IRES cu link-ul: http://www.ires.com.ro/uploads/articole/ires_obiceiurile_alimentare_ale_romanilor_2013.pdf

**Fig. 7:**
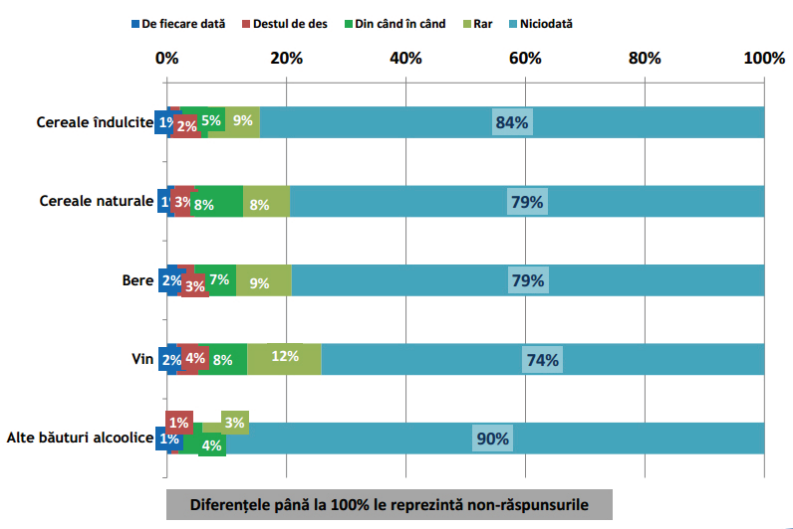
Incidența de consum a grupelor alimentare meționate în grafic în timpul Cinei (Continuare). Reprodus de pe pagina IRES cu link-ul: http://www.ires.com.ro/uploads/articole/ires_obiceiurile_alimentare_ale_romanilor_2013.pdf

Deși sondajul IRES nu precizează culoarea pâinii, luând în considerare că pâinea albă este în medie mai ieftină decât painea neagră iar peste 70% din populație explică că motivul principal pentru care nu se alimentează sănătos o reprezintă motivul financiar [**10**], se poate deduce ușor că cel mai des consumat tip de pâine consumată este pâinea albă. După cum se poate observa din figurile, pâinea este alimentul preferat în timpul micului dejun, prânzului, și cinei; dar, consumul zilnic de pâine albă este un factor favorizant pentru dezvoltarea diabetului zaharat tip 2 [**23**]. La răndul său, diabetul zaharat poate duce în timp la HA. 

În continuare, atât cafeaua cât și ceaiul conțin substanța numită cofeină. Cofeina are efecte de creștere a tensiunii sistolice și diastolice ajungând treptat până la valori de HA [**24**-**28**]. 

În continuare, se poate observa că 3 din cele 5 alimente și băuturi cele mai des consumate în timpul micului dejun au un risc direct de dezvoltare în timp a HA. Deci, se poate ușor deduce că există o relație directă de cauzalitate între numărul crescut de cazuri de HA în România, comparativ cu celelalte state membre ale UE și regimul alimentar al populației.

**Ipoteze privind mecanismele producerii de HA din cauza dietei nesănătoase**

Cauzele HA sunt numeroase, însă pentru scopul acestui articol vor fi discutate doar cazurile de HA primară care au ca factor etiologic consecințele unei diete nesănătoase, și anume: (1) diabetul zaharat provocat de către aportul excesiv de alimente ce conțin zahăr sau făină albă (2) responsivitatea crescută la catecolamine a persoanelor sensibile la sare dată de aportul crescut de alimente ce conțin cantități mari de NaCl, și (3) ateroscleroza provocată de aportul crescut de alimente ce conțin cantități mari de lipide [**1**,**16**].

**Diabetul Zaharat și HA**

Diabetul zaharat și HA arterială au coexistat permanent ca afecțiuni care își amplifică simptomatologia reciproc. Prezența HA și a diabetului zaharat la același individ, cresc semnificativ mortalitatea și este estimat că 35-75% din complicațile diabetului sunt cauzate de către HA [**29**-**32**]. Mecanismul prin care diabetul zaharat produce HA este cel mai probabil creșterea rezistenței vasculare prin distrugerea parțială a endoteliului vascular [**29**,**33**-**35**].

De asemenea, o urmare devastatoare a diabetului zaharat de tip 1 sau 2 o constituie instalarea nefropatiei diabetice [**29**,**36**]. Numeroase studii arată că pe măsură ce nefropatia diabetică avansează, HA se agravează [**29**].

**Aportul Crescut de Sare și HA**

Consecințele excesului de sodiu ingerat includ modificări funcționale prezente atât în exteriorul celulelor cât și în interiorul acestora. Astfel, la un nivel crescut de Na în sânge, apa difuzează pasiv de la nivelul celulelor endoteliale în mediul extracelular și crește volumul sanguin circulant astfel producând hipervolemie. Hipervolemia reprezintă un factor de facto pentru dezvoltarea HA [**7**,**16**,**20**,**21**].

În continuare, la nivelul mediei vaselor sangvine crește tonusul musculaturii netede vasculare și crește sensibilitatea sa față de catecolamine și angiotensină care sunt substanțe vasoconstrictoare. Această sensibilitate crescută la vasoconstrictoare duce la rigidizarea vaselor sangvine care vor duce la HA [**7**,**16**,**20**,**21**].

Un al treilea mecanism prin care aportul crescut de sare din dietă poate duce la HA este prin acumularea de Na+ în zonele baroreceptoare. Această acumulare va duce larigidizarea pereților arteriali în aceste zone și astfel se va înregistra o scădere a sensibilității baroreceptorilor. Ulterior, prin scăderea sensibilității baroreceptorilor se va diminua abilitatea reglatorie a tensiunii arteriale a baroreceptorilor [**7**,**16**,**20**,**21**].

Toate aceste mecanisme solitare sau combinate prezintă o platformă declanșatoare pentru evoluția HA care poate duce treptat la alte afecțiuni cardiovasculare. 

**Beneficiile reducerii cantitații de sare alimentară (NaCl) din alimentație**

Beneficiile reducerii aportului de sare în alimentația zilnică includ (1) reducerea directă a tensiunii arteriale (2) reducerea riscului de moarte prin atac cerebral (3) revenirea la valorile normale ale dimensiunilor cardiace crescute datorită hipertensiunii arteriale (4) reducerea riscului apariției litiazei renale. 

**Aportul crescut de alimente ce conțin cantități mari de lipide și HA:**

Consumul alimentelor bogate în grăsimi cresc riscul dezvoltării aterosclerozei (ATS). La rândul său, ATS reprezintă o afecțiune caracterizată prin depunerea plăcilor de aterom în interiorul vaselor sanguine care produc obstruția parțială a vasului [**22**].

Cu cât cavitatea din interiorului vasului este mai mică, cu atât presiunea arterială în vas crește; astfel, în timp se poate ajunge la HA [**22**]. În cazuri severe de HA cuplată cu factori de stres, o mică porțiune din placa de aterom se poate desprinde din locul de depunere și poate circula până la nivelul arteriolelor cerebrale sau vaselor coronariene producănd ocluzia acestora. Datorită ocluziei, se poate instala infarctul miocardic acut sau accidentul vascular cerebral care poate evolua spre paralizie parțială, deficite cognitive și chiar deces [**21**-**22**].

Există mai mulți factori de risc pentru dezvoltarea ATS printre care se numără (1) hiperlipidemia dată de aportul crescut de grăsimi (2) HA și (3) diabetul zaharat [**22**]. Din câte se poate observa, toți acești factori pot fi corectați prin introducerea unui regim alimentar sănătos.

**Ce reprezintă o dietă sănătoasă?**

Dieta sănătoasă se referă la adoptarea obiceiurilor de alimentație în urma cărora o persoană începe să simtă treptat o stabilizare a stării de spirit și trăiește continuu o stare de plinețe de energie [**19**]. De asemenea, dieta sănătoasă are drept scop îmbunătățirea stării de sănătate și menținerea ei atât pe termen scurt cât și pe termen lung. Menținerea sănătății prin învățarea unor elemente de nutriție de bază și utilizarea lor într-un mod care funcționează eficient reprezintă la momentul actual cea mai eficientă formă de prevenție a numeroase afecțiuni printre care se numără și HA [**1**-**4**,**7**,**17**-**19**].

De asemenea, este important de menționat că dieta sănătoasă nu reprezintă neapărat o filosofie nutriționistă strictă formată din idei nerealiste de slăbire; mai mult, alimentația sănătoasă nu are drept scop autodeprivarea de alimentele favorite [**19**]. Din contră, o dietă sănătoasă are drept scop îmbunătățirea sănătății fizice și psihice, iar autodeprivarea de alimentele favorite pe termen lung poate duce la diminuarea sănătății psihice. Astfel dieta sănătoasă reprezintă o combinație dintre alimentele favorite și alimentele cu rol nutrițional.

**Instrumente utile în crearea unui regim alimentar sănătos**

Pentru a crea un regim alimentar sănătos, Universitatea Harvard din Massachussets, S.U.A., a înaintat o bază de date întitulată “nutrion score” care poate fi folosită de către clinicieni, nutriționiști și publicul general pentru a crea o dietă personalizată formată din alimente gustoase și sănătoase [**4**]. Pagina web a acesteia poate fi găsită în bibliografie și include mai multe informații privind formarea unei diete personalizate cât și articole privind prevalența diabetului la tineri, efectul diabetului asupra generațiilor viitoare, efectul NaCl asupra bolilor cardiovasculare și alte resurse folositoare [**4**,**7**-**9**].

**Alimentele recomandate**

Un regim alimentar sănătos cu un risc redus de dezvoltare a HA include alimente precum carnea slabă degresată. Acest tip de carne include carnea de vită, vițel, carne de pasăre, pui, fazan și curcă, toate consumate fără piele [**14**]. Este de preferat ca mielul și porcul să fie consumate mai rar de o dată pe săptămână [**14**]. 

Alte alimente sanogene includ: (1) peștele; (2) produse lactate precum lapte degresat, iaurt slab, branză degresată și (3) toate tipurile de fructe și legume [**14**].

**Alimentele nerecomandate**

Printre alimentele nerecomandate sunt incluse carnea grasă precum carnea de oaie, coasta de porc, rață, și gâscă [**14**]. Alte alimente nerecomandate includ lactate precum laptele nedregresat concentrat, iaurt nedegresat, și brânzeturi cu mai mult de 50% grăsimi [**14**].

Ca mod de preparare, alimentele fierte sunt în mare parte mai sanogene decât cele prăjite. Astfel, alimentele prăjite nu sunt recomandate. De asemenea, pentru prevenția HA nu este recomandat consumul exagerat de cafea sau ceai.

**Concluzie**

În urma comparării incidenței HA la populația din România cu regimul alimentar al acesteia, se poate observa o legătură directă între modul de alimentație și numărul cazurilor de HA. Mecanismele principale de dezvoltare a HA datorită unui regim alimentar nesănătos includ apariția diabetului zaharat ca urmare a aportului crescut de zahăr și făinoase, aportul crescut de NaCl, sau ateroscleroza dată de aportul crescut de lipide. Pentru a reduce numărul de cazuri de HA și pentru a reduce riscul de complicații în urma acesteia este vitală introducerea unui regim alimentar sănătos. 
